# *In Vivo* Identification of Solar Radiation-Responsive Gene Network: Role of the p38 Stress-Dependent Kinase

**DOI:** 10.1371/journal.pone.0010776

**Published:** 2010-05-21

**Authors:** Nicolas Mouchet, Henri Adamski, Régis Bouvet, Sébastien Corre, Yann Courbebaisse, Eric Watier, Jean Mosser, Christophe Chesné, Marie-Dominique Galibert

**Affiliations:** 1 CNRS UMR 6061 Institut de Génétique et Développement de Rennes, Equipe RTO, Rennes, France; 2 Université de Rennes 1, IFR140 GFAS, Faculté de Médecine, Rennes, France; 3 PROCLAIM, Saint Grégoire, France; 4 CHU Rennes, Service de Dermatologie, Rennes, France; 5 CHU Rennes, Laboratoire de Génomique Médicale, Plateforme Transcriptomique GenOuest, Rennes, France; 6 CHU Rennes, Service de Chirurgie Plastique, Rennes, France; University of Missouri-Kansas City, United States of America

## Abstract

Solar radiation is one of the most common threats to the skin, with exposure eliciting a specific protective cellular response. To decrypt the underlying mechanism, we used whole genome microarrays (Agilent 44K) to study epidermis gene expression *in vivo* in skin exposed to simulated solar radiation (SSR). We procured epidermis samples from healthy Caucasian patients, with phototypes II or III, and used two different SSR doses (2 and 4 J/cm^2^), the lower of which corresponded to the minimal erythemal dose. Analyses were carried out five hours after irradiation to identify early gene expression events in the photoprotective response. About 1.5% of genes from the human genome showed significant changes in gene expression. The annotations of these affected genes were assessed. They indicated a strengthening of the inflammation process and up-regulation of the JAK-STAT pathway and other pathways. Parallel to the p53 pathway, the p38 stress-responsive pathway was affected, supporting and mediating p53 function. We used an *ex vivo* assay with a specific inhibitor of p38 (SB203580) to investigate genes the expression of which was associated with active p38 kinase. We identified new direct p38 target genes and further characterized the role of p38. Our findings provide further insight into the physiological response to UV, including cell-cell interactions and cross-talk effects.

## Introduction

The skin is a complex organ composed of organized cells that constitute a unique physiological barrier. The skin has a diverse range of protective functions in the face of a number of biological, chemical and physical hazards, including UV-radiation.

The sun's radiation that reaches Earth contains UV made up of a combination of UVA (95%) and UVB (5%). The cytotoxicity induced by these UV rays depends on their wavelength. UVA (320–400 nm) penetrates deeply, as far as the dermis, generating reactive oxygen species (ROS), including superoxide radicals, hydrogen peroxide and hydroxyl radicals. These ROS, in turn, lead to changes in protein and DNA [1]–[4]. UVB radiation (280–320 nm) mainly affects cells of the epidermis layer, with only 10 to 15% of the radiation penetrating the dermis. UVB rays cause direct DNA damage, producing pyrimidine dimers and pyrimidine-pyrimidone photoproducts [5]. UV is considered to be the agent that causes most damage to DNA, contributing to skin aging, photodermatoses and carcinogenesis.

Cells have developed multiple mechanisms to mitigate the effects of UV exposure, including specific photo-protective responses involving increased production of melanin pigment, the most efficient UV-absorbing agent [6]. Dedicated DNA-repair machineries are also activated and nucleotide excision repair (NER) protein complexes are recruited to remove DNA photolesions [7]. UV radiation mediates a variety of additional cellular reactions, including inflammation and cell-cycle regulatory events [8], [9].

Inflammation is mostly due to UVA [10]. UVA induction of inflammation entails a cascade of early events involving the infiltration of inflammatory blood leucocytes four to six hours after irradiation, increased production of prostaglandins, release of TNF-alpha and inflammatory cytokines and activation of the NFKB transcription factor pathway [11]. Cell-cycle arrest and apoptosis of cells are also seen in response to UV. The occurrence of these two cellular responses depends on the amount of UV to which cells are exposed. Phosphorylation and stabilization of the p53 tumor suppressor involves the Mitogen Activated Protein kinase (MAPK) p38α and the ATM/ATR pathway [12], [13]. MAPK p38α induces the transcriptional activity of p53, leading to cell-cycle arrest, DNA repair and apoptosis [14]. Low UV doses induce transient cell-cycle arrest by up-regulating p53 target genes and thereby eliciting DNA repair processes [15]. High UV doses induce p53-mediated apoptosis through the up-regulation of p53 target genes [15]. The apoptotic response is also mediated by the stress-activated p38 kinase (MAPK14), which modulates p53 activity through TP63 phosphorylation [16]. The activity of the downstream target of p38, USF-1, is modulated through post-translational modification, again depending on the nature and dose of UV exposure, either promoting gene expression or inducing a transcriptional block [17], [18].

The response to UV is complex, affecting both transcription and protein activity. Several human *in vitro* models have been developed to facilitate studies of the molecular processes involved, and in particular focusing on pigmentation [19], [20], DNA-repair or inflammation [21]. These models include mono-cultured cell lines, co-cultured melanocytes and keratinocytes, and reconstructed epidermis. Low doses of UVB radiation have been shown to cause a greater increase in melanin synthesis in human co-culture models based on melanocyte and keratinocyte cells isolated from the foreskin or from skin donors of various phototypes [22], [23] than in mono-cultured melanocytes [23], [24]. This highlights the cooperative role of keratinocytes and melanocytes. Similar findings have been obtained for inflammation [25]. An *in vitro* three-dimensional model of reconstructed human skin epidermis has been developed to study cooperation between keratinocytes and melanocytes. However, the model does not take into account the different cell types, cell ratios or cellular organizations. Indeed, in the physiological epidermal structure, melanocytes lie over the basal membrane, surrounded by keratinocytes and protected by the stratum cornea. This specific architecture may affect the cellular response to UV radiation. We therefore further investigated the UV-induced response by studying gene expression profiles in the epidermis using skin samples exposed, *in vivo*, to simulated solar radiation, containing environmentally relevant amounts of UVB and UVA. A whole-genome approach was used to investigate expression profiles. We also used *ex vivo* assay in the presence of a specific p38 kinase inhibitor to identify among the differentially expressed genes, those for which the transcription was specifically mediated through the stress-activated p38 kinase.

## Results

### Transcriptional profile following *in vivo* simulated solar radiation (SSR)

**Figure 1:**
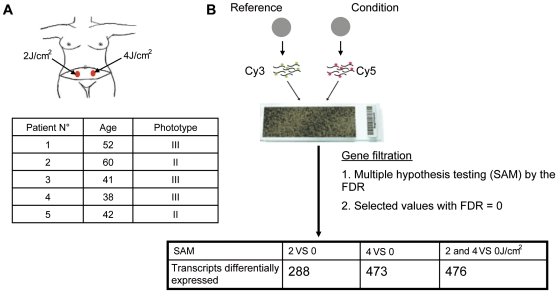
Experimental design. (A) Simulated solar irradiation (SSR) of abdominal areas, at doses of 2 and 4 J/cm^2^, five hours before plastic surgery. Five women with a median age of 42 years, with phototype of II or III according to Fitzpatrick classification, were included in a photobiological study. (B) Microarray workflow. Non-irradiated and irradiated samples were separately compared to a reference, containing pooled samples (non-irradiated and irradiated). Significance Analysis of Microarrays (SAM) and a false discovery rate (FDR) of zero were used to identify transcripts that were differentially expressed between irradiated and non-irradiated conditions. SAM comparing 2 J/cm^2^ with non-irradiated samples identified 288 differentially regulated genes; comparison between 4 J/cm^2^-irradiated and non-irradiated samples identified a set of 473 differentially regulated genes; and 3-class SAM identified a set of 476 genes (Table S1).

**Figure 2:**
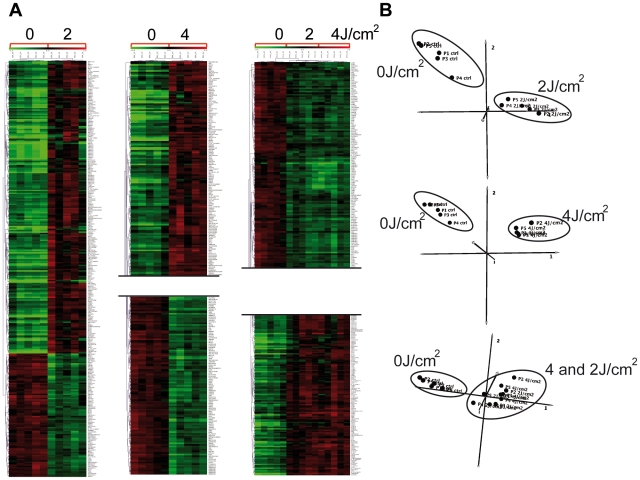
Gene and sample classification analyses. Hierarchical Clustering (HC) (A) and Principal Component Analysis (PCA) (B) (Mev 4.4) were used to classify the differentially expressed genes after SAM normalization. The normalized expression for each gene (rows) in each sample (columns) is presented with a color code (green for down-regulated and red for up-regulated). Each HC analysis (A) formed groups confirmed by PCA (B), showing clearly distinct clusters for UV-irradiated and control samples.

**Figure 3:**
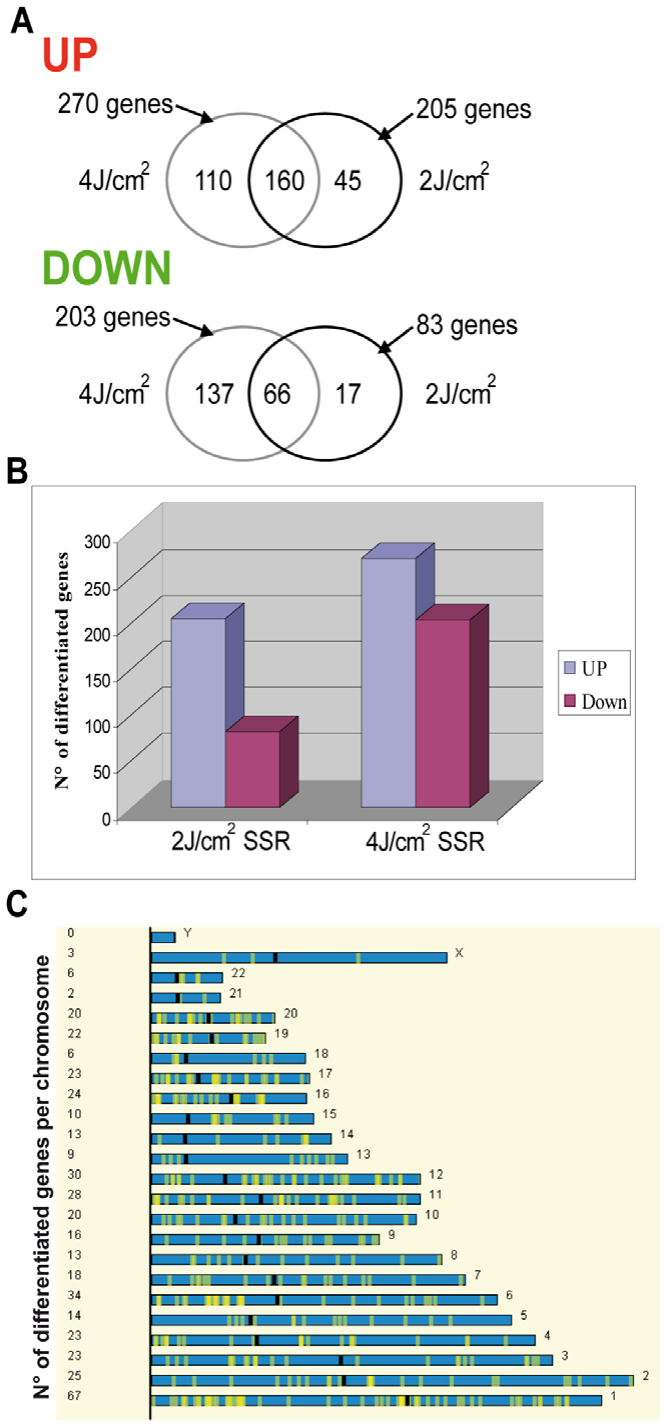
Gene distribution as a function of UV treatment and chromosome location. The Venn diagram (A) shows up- and down-regulated genes following 2 J/cm^2^ and 4 J/cm^2^ SSR. The diagram shows the number of genes in each group (UP or Down). The histogram (B) shows differentially expressed genes, demonstrating variation in response as a function of dose. The number of down-regulated genes is greater for the higher dose. Differentially regulated genes in response to UV exposure (C) do not show clustering for chromosomal location (the number of UV regulated genes is reported in Table S2).

To examine the effects of solar radiation on gene expression *in vivo*, we carried out a photobiological study in five healthy volunteers referred for abdominal plastic surgery. Volunteers were aged between 38 and 60 years, with a median age of 46. Skin phototypes were determined by detailed interview and using the sun-reactive skin type classification [26]. Two patients were classified as skin phototype II and three as skin phototype III (Fig. 1A). Five hours before resection of the pre-defined abdominal skin region, two areas were irradiated with a solar simulator at doses of 2 and 4 J/cm^2^, as shown in Figure 1A. Gene expression profiles were obtained for irradiated and non-irradiated samples using whole-genome arrays (Agilent 44K) (Fig. 1B). Array data were normalized and scaled using R software (http://www.bioconductor.org). We then used the Significant Analysis of Microarray (SAM) statistical method to identify genes for which expression was modified by SSR. Pair-wise comparisons were made between non-irradiated and irradiated skin samples to analyze the effect of irradiation on gene expression. Two-class SAM identified a set of 288 genes, the expression of which differed between samples exposed to 2 J/cm^2^ and non-irradiated samples, and a set of 473 genes, the expression of which differed between samples exposed to 4 J/cm^2^ and non-irradiated samples. These genes accounted for about 1.5 to 2.5% of the “valid” genes tested. Hierarchical Clustering (HC) of these genes grouped together samples for each condition in a single branch and highlighted two gene clusters, named UP and DOWN, according to their relative expression level (Fig.1B-2A). Three-class SAM analysis of 2 J/cm^2^, 4 J/cm^2^ and non-irradiated conditions gave a set of 476 differentially expressed genes. HC grouped the two groups of irradiated samples together in a single branch and the non-irradiated samples in a separate branch and identified two gene clusters. Principal Component Analysis (PCA) confirmed segregation of irradiated and non-irradiated samples, whereas no segregation between the two groups of irradiated samples was detected, consistent with the HC (Fig. 2B). We next compared the genes included in UP-clusters and DOWN-clusters. Use of a Venn diagram revealed that 160 genes were grouped as “UP” and 66 genes as “DOWN” for both 2 J/cm^2^ and 4 J/cm^2^ irradiated samples (Fig. 3A, Table S1); these genes corresponded to 80% of all genes found to be differentially expressed in 2 J/cm^2^ irradiated samples. The number of down-regulated genes increased in a UV dose-dependent manner (Fig. 3B), consistent with the notion that increasing the dose of UV increases DNA damage, causing more cell arrest and apoptosis, which in turn reduces gene transcription overall.

### Chromosomal location of differentially expressed genes

To investigate whether there is a UV-responsive gene cluster, we used Onto-Express (http://vortex.cs.wayne.edu/projects.htm) to determine the chromosomal location of differentially expressed genes following SSR exposure. No UV-responsive clusters were identified (Fig. 3C). UV-regulated genes were distributed randomly among chromosomes. The percentage of genes per chromosome that were found to be UV-regulated was between 0.20% (chr X) to 1.38% (chr 3), for an SSR dose of 2 J/cm^2^, and 0.15% (chr X) to 2.56% (chr4) for 4 J/cm^2^ (Table S2). Chromosomes 21, 22 and X displayed the lowest percentages for both doses used (Table S2).

### Functional annotation

To investigate the biological and molecular processes and pathways affected in response to SSR, we analyzed the list of differentially expressed genes using EASE (Expression Analysis Systemic Explorer, http://david.abcc.ncifcrf.gov/tools.jsp). This online software package identifies groups of biologically related genes. We focused on significant gene sets belonging to three GO categories: biological processes, molecular function and cellular components. Eighty % of the annotated transcripts showing differential expression upon UV exposure at 2 J/cm^2^ also showed differential expression at a dose of 4 J/cm^2^. Thus, most (80%) of the biological processes (GO terms) implicated by genes that were differentially expressed at 2 J/cm^2^ exposure were also highlighted by genes differentially expressed at 4 J/cm^2^. Additional genes showing differential regulation upon 4 J/cm^2^ exposure only were associated with other GO terms, representing almost 60% of these UP- and Down-regulated gene sets (Table S3). The GO terms common to both the 2 and 4 J/cm^2^ SSR-regulated gene sets highlighted two fundamental biological processes: inflammation and apoptosis. Inflammation was associated with the set of UP-regulated genes and is known to be the most common and immediate response to UV-induced damage. Solar radiation elicits the production of chemokines inducing cytokine activity, leading to cell-cell communication within both the dermis and the epidermis. The main inflammatory responses identified here included the promotion of cell proliferation, cell development and keratinocyte differentiation. This suggests that, despite the negative effects of SSR, the skin epidermis increases its thickness to protect against additional damage. Consistent with this, the associated cellular component was the extra-cellular space and the nucleus. Events associated with apoptosis and the regulation of apoptosis were found in both the UP- and Down-regulated gene sets, together with processes that concomitantly regulate the cell cycle and protein kinase activity (both cyclin dependent and independent). KEGG pathway analysis (DAVID analysis) identified only the p53-signaling pathway (hsa04115) as being involved in the SSR-induced events common to both sets of genes.

**Figure 4:**
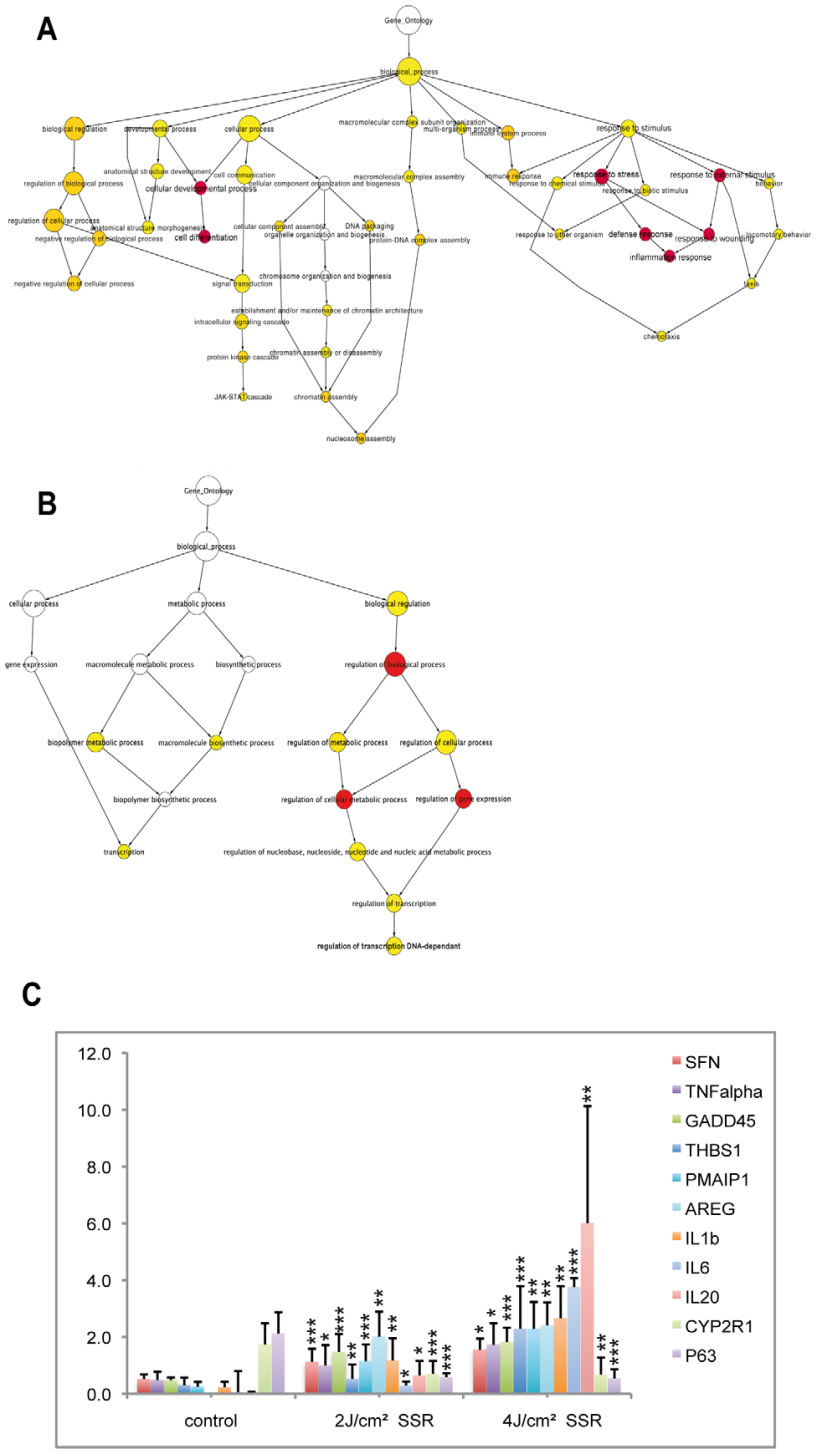
GO tree for genes showing differential regulation only with a dose of 4 J/cm^2^ SSR and qPCR validation. Genes showing differential regulation only in response to the higher dose of SSR are annotated with several GO terms. Up-regulated genes (A) were associated with terms that included negative regulation of cellular processes, morphogenesis of anatomical structures, JAK-STAT signaling, nucleosome assembly and the immune response (inflammatory response and cell differentiation were also found for 1 MED (minimal erythema dose: 2 J/cm^2^), as indicated in red); for down-regulated genes (B), GO terms were predominantly associated with transcription and regulation, although three GO terms (in red) relating to “regulation of transcription” were also observed for 1 MED (Table S3). (C) Microarray data were validated for 11 genes (nine up and two down-regulated): mRNA was assayed by qPCR and normalized to the values for 18S mRNA. These genes were selected from the list of differentially expressed genes, for their classification as inflammatory response genes or their involvement in the p53 pathway. Error bars represent standard deviation. Stars indicate significant differences (two-tailed Student's t-test) between control and irradiated samples: * P<0.05; ** P<0.01; *** P<0.001.

We then analyzed the GO terms associated with the set of genes that showed differential regulation only for high-dose SSR exposure (Fig. 4). These genes were associated with additional processes and indicated a further up-regulation of the inflammatory response. Indeed, the proportion of up-regulated genes that were annotated with GO terms associated with inflammation, cell development and cytokine activity were considerably higher for the higher SSR dose (52%, 44% and 50%, respectively) than for lower dose responses. At this higher dose, there was evidence of the involvement of new processes, particularly the immune response, chemotaxis and JAK-STAT cascade activation (Fig. 4A). Consistent with this, KEGG pathway analysis showed the involvement of the p53 signaling pathway and cytokine-cytokine receptor interaction (hsa04060) in the response to the higher dose. This reflects the important roles of the kinase and cytokine activities in the underlying molecular mechanisms. We also observed transcriptional and transcriptional regulatory events associated with the down-regulated gene set, suggesting a reduction in transcription to favor the damage response (Fig. 4B). These findings are evidence of the activity of a variety of processes in the epidermis, eliciting different responses according to the degree of radiation.

### Validation of photo-induced expression by qRT-PCR

Real-time PCR was used as an independent method to validate microarray expression data and to analyze quantitative gene expression data. Eleven genes were selected (nine up-regulated and two down-regulated). The nine up-regulated genes were chosen as belonging to the p53-signaling pathway or to the cytokine-cytokine receptor interaction process. The two down-regulated genes were chosen for their involvement in vitamin D3 hydroxylation (*CYP2R1*) and in epithelial regenerative proliferation (*TP63*, a *p53* homolog). Gene expression assays were performed using the same sample set for technical validation and five additional samples (from five different individuals) for biological validation. The two sample sets gave similar expression profiles in response to SSR, thus validating the data (Fig. 4C shows mean values for total expression data for the two sample sets). Quantitative RT-PCR results were statistically significant and were consistent with microarray data. Genes up-regulated in response to SSR remained in the UP set in both samples sets, and down-regulated genes remained in the DOWN set. However, although gene expression profiles for different skin samples were similar, the extent of changes in expression differed, as reflected by the error bars for mean expression data. This suggested inter-individual variation, independent of skin phototype. Gene expression profiles differed as a function of SSR dose and correlated with the microarray data. The radiation-induced response observed for the tested genes was higher at higher dose. Indeed, the *IL6* and *IL20* mRNA responses were stronger with 4 J/cm^2^ than with 2 J/cm^2^. *IL20* expression levels were 52 times higher in samples exposed to low-dose (2 J/cm^2^) radiation and 490 times higher in samples exposed to high-dose (4 J/cm^2^) radiation than in non-irradiated samples. In comparison, *SFN (stratifin), TNF-α* and *GADD45α* mRNA levels were 2 to 3 times higher in 2 J/cm^2^ irradiated samples than in samples from non-irradiated areas, but were not higher still in samples irradiated with 4 J/cm^2^ (3 to 3.7 times higher than in non-irradiated samples). We also observed the down-regulation of *CYP2R1* and *TP63* in samples irradiated with a dose of 2 J/cm^2^, with levels 2.4 and 3.7 times lower, respectively, than in non-irradiated samples and remaining low in samples irradiated with the 4 J/cm^2^ dose. Taken together these results validate the microarray data.

### A role for p38 in the response to SSR: Gene network analysis and skin explants

**Figure 5:**
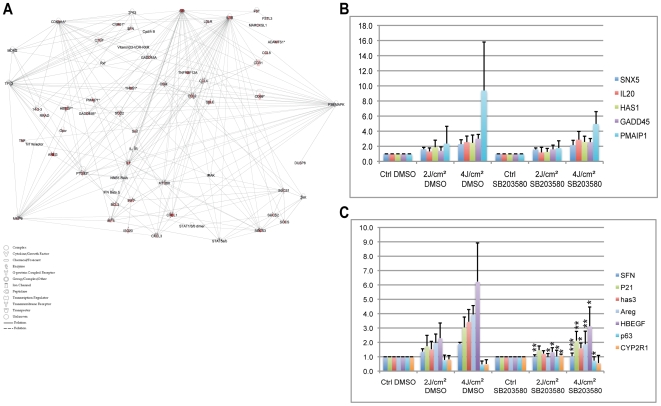
The p38 pathway network for the 4 J/cm^2^ SSR response. Identification of p38-dependent genes *ex vivo*. *Ex vivo* study of the p38 pathway (A) in response to SSR using Ingenuity Pathway Analysis. Edges of the diagram are labeled with a description of the relationship between the nodes. Lines between genes represent known interactions, with solid lines representing direct interactions and dashed lines representing indirect interactions. Nodes are displayed using various shapes that represent the functional class of the gene product (see legend). Genes showing up-regulation of expression levels in response to SSR are in red. Skin samples from patients participating in the *in vivo* study were used for *ex vivo* analysis, with the same conditions as used *in vivo*. Genes belonging to the p38 pathway were identified using the specific kinase inhibitor SB203580. Genes not regulated by p38 are shown in B and those regulated by p38 are shown in C. Error bars represent standard deviations. Stars indicate significant differences (two-tailed Student's t-test) between DMSO-treated and SB203580-treated samples. * P<0.05; ** P<0.01; *** P<0.001 (Table S4). Values for irradiated DMSO and SB203580 samples were significantly different to values for their non-irradiated controls (data not shown).

The p53 pathway is a major and well-characterized pathway that is switched on following radiation. However, additional kinase processes including the stress responsive p38 kinase may also mediate UV- and solar radiation-induced responses. To identify downstream targets of p38 in the response to solar radiation, we used Ingenuity Pathway Analysis software (IPA) to identify potential links between genes that were differentially regulated in response to high-dose radiation and the p38 stress kinase (Fig. 5A). We analyzed all the networks generated by IPA, based on published databases, merging those that include p38 to sequentially enrich for p38-related genes. About half of the retrieved genes were associated with the p53-signaling network and half were associated with the cytokine-cytokine receptor network. The interactions identified, involving either the genes or proteins, were mainly indirect (dotted lines, Fig. 5). We therefore set up an *ex vivo* assay to identify the genes for which changes in expression could be linked directly to activation of the p38 pathway, using SB203580, a p38-specific inhibitor [27]. We also screened for new genes for which UV-dependent transcript levels could be linked to the p38 pathway. Skin punch biopsy samples were taken from six abdominal plastic surgery resections, three of which were from skin donors used for the experiments described above, and cultured in the presence or absence of a p38-specific inhibitor. Protocols for SSR and time of sample collection were the same as for the *in vivo* assays. Quantitative RT-PCR experiments were carried out for genes that were in either the UP or Down clusters, and for which an indirect relationship with the p38 pathway had been identified (*HAS1, GADD45α, SFN, CDKN1A*) (Fig. 5A). We also tested various genes for which a relationship with p38 can be suspected (*SNX5, IL20, PMAIP1, AREG, HAS3, HBEGF, p63, Cyp2R1*). Gene expression profiles obtained for irradiated skin biopsy samples were comparable with *in vivo* data, and UV-induced changes in expression levels were statistically significant (Fig. 5B–C). However, the magnitudes of the changes in mRNA abundance were smaller than in *in vivo* experiments, consistent with previous reports [27]. To investigate whether UV-induced changes in gene expression were dependent on the p38 pathway, we used the statistical t-test comparing UV-induced gene expression levels in the presence and absence of the inhibitor SB203580. The t-test split the genes into two groups, p38-independent (genes for which differences in expression levels in the presence and absence of SB203580 were not significant; Fig. 5B) and p38-dependent (genes showing a significantly smaller UV-induced response in samples treated in the presence than absence of SB203580 Fig. 5C and Table S4). This approach thus allowed us to determine the nature of the relationship between p38 and various genes (*SFN, CDKN1A*) and to identify *HAS3, HBEGF, AREG* as new physiological p38 targets, *AREG* and *HBEGF* being part of the inflammatory response.

## Discussion

Using *in vivo* whole-genome transcript profiling, we quantified the effect in the epidermis of environmentally relevant levels of solar radiation on the expression of genes that mediate cellular responses to UV, the most common and damaging threat to human skin. Using the doses and gene-expression filters described, we found that in the epidermis of Caucasian skin irradiated *in vivo* about 1.5% of the genome is modulated in a UV dose-dependent manner. Genes differentially expressed in non-irradiated and SS-irradiated skin areas included both UP- and Down-regulated genes. These genes were randomly distributed among chromosomes, consistent with the absence of any substantial UV-controlled regions.

The use of gene annotation coupled to this *in vivo* approach allowed the global response of the skin epidermis to be studied as it occurs in real life, including the effects of paracrine mediators secreted by the dermis layers and the coordinated responses of epidermal cell populations. Indeed, this approach preserves interactions between cells present in the correct ratios, with physiological organization of the epidermal 3D-structure supported by the dermis. Keratinocytes are the predominant epidermal cell sub-population, but melanocytes, fibroblasts and other cell types, also known to participate individually in the solar radiation response, are present as in natural conditions [28]–[30]. Importantly, the differentiation state of cells in the epidermis changes over time [31]. Keratinocytes undergo a complex and precise program of differentiation; they start at the basal layer of the epidermis where they proliferate and move on to the stratum corneum. The nature of the biological processes taking place, including the response to UV, would thus be expected to depend on their differentiation state. This issue needs to be incorporated into any accurate description of the response, as it is the case in our model. Also, unlike data from monolayers of cultured cells, our model reveals the effects of radiation taking into account the ability of the wavelengths to penetrate through the stratum corneum before hitting the epidermal and dermal cell layers. Indeed, UVA and UVB rays penetrate the skin to different extents and elicit distinct pathways, dependent not only on the doses delivered but also on their ability to generate direct or indirect DNA photolesions [9]. Previous studies have focused on the effects of either UVA [32], [33] or UVB [34]–[40] on skin or cell lines. Our findings, however, reflect the effects of both types of UV, at a dose of 2 J/cm^2^ corresponding to the minimal erythemal dose for the skin of Caucasians. The specific responses of particular sub-populations of skin cells (for example melanocytes or Langherhans cells) cannot be quantified in our analyses due to filters that we applied. Nevertheless, this global approach importantly integrates cell-cell cross-talk mediated by paracrine factors, secreted within the epidermal and the dermal cell layers of the skin [41]. Additionally, due to the combination of both UVA and UVB in environmentally relevant proportions and doses, the epidermis transcriptome addressed in our model reflects the general biological response to sun exposure with minimal bias.

To analyze the cell processes that are activated early following environmentally relevant levels of radiation, we examined gene expression profiles five hours after SSR, delivered at doses of 2 J/cm^2^ and 4 J/cm^2^. Two sets of differentially expressed genes were identified after SS irradiation, defined as UP and Down gene clusters. Analysis of the number of genes differentially expressed in response to radiation at the two different doses was consistent with the idea that gene expression levels are dependent on radiation dose [29], [36], [40], [42]. However, whereas the number of down-regulated genes was 2.5 times higher in samples exposed to a dose of 4 J/cm^2^ than in samples irradiated at 2 J/cm^2^, the number of genes present in the UP clusters was generally similar at the two doses (only 1.3 times more in the higher dose samples). In addition, about 80% of the genes grouped in either the UP or Down clusters following minimal SSR dose exposure were also differentially expressed following irradiation at a dose of 4/Jcm^2^ (59% of genes from the UP gene set and 33% of the Down gene set). PCA diagrams confirmed this observation, indicating that responses differed between irradiated and non-irradiated samples, but that the responses observed for irradiated samples were hardly distinguishable between the two different doses. It is possible that the responses induced by the lower dose form part of the response observed following exposure to the higher dose. Indeed, the response observed for 2 J/cm^2^, which corresponds to the minimal erythemal dose in our study, could be considered as a basal or sub-acute response and the 4 J/cm^2^ as an acute dose for Caucasian skin.

Functional annotation based on gene ontology and enrichment for GO terms allowed identification of the molecular processes stimulated in the skin epidermis in response to solar radiation. The protective response in the epidermis was represented by two major GO terms observed for gene sets identified for both irradiation doses: cell differentiation and the inflammatory response. Cell differentiation is associated with the subsequent thickening of the skin resulting from both epidermal hyperplasia and increased thickness of the stratum corneum in response to UV light [44]. The enrichment of genes involved in inflammation was observed with the high irradiation dose. These genes included those encoding chemokine family members and their receptors (*TNFα*, *CCR1, CXCL3, CCL2, CCL4, CCL8, OSM,* and *RELT*). Through KEGG pathway analysis, these genes suggested the involvement of a cytokine-cytokine receptor interaction pathway, implicating cell-cell communication in UV response mechanisms. Suppressor of cytokine signaling (SOCS) proteins and the JAK-STAT pathway are also involved in the tight regulation of the inflammation process. SOCS proteins are intracellular cytokine-inducible proteins that prevent JAK-mediated activation of STAT3, a crucial mediator of inflammatory processes, from attenuating cytokine signaling. *SOCS3* was up-regulated in response to both radiation doses. However, *SOCS1* and *SOCS2* were significantly up-regulated only in samples exposed to 4 J/cm^2^ radiation, presumably to balance cytokine signaling further and moderate the acute inflammatory response. The production of reactive oxygen species (ROS) induced by both UVA and UVB also contributes to inflammation, by activating the JAK/STAT pathway. Oxidative stress generated by free radicals modulates tyrosine phosphorylation of JAK and activates the translocation of STAT dimers to the nucleus, where they transactivate cytokine target genes [43]. UV light thus activates inflammation, at the same time stimulating cell survival mechanisms, cell proliferation and cell differentiation. These findings provide the first *in vivo* evidence of the involvement of the JAK-STAT pathway —strengthening the immune response— in skin photo-biology, and support previous observations [34], [36]-[38], [45]. Our findings also show the importance of analyzing the global effects induced by solar radiation.

Under UV-irradiation, the cell-cycle process is tightly regulated to allow repair of DNA photolesions, to resume the cell-cycle or to direct cells towards apoptosis. The set of up-regulated genes showed a particularly strong link to p53, which promotes cell-cycle arrest, together with *CDKN1A* [46], *SFN* [47] and *GADD45* [48]. Indeed, the cyclin-dependent kinase inhibitor *CDKN1A*, a potent inhibitor of several cyclin-dependent kinase complexes (*CyclinD/CDK4/6; CyclinB/CDK2*) mediates G1 arrest, whereas *SFN* and *GADD45* inhibit the *CyclinB/Cdc2* complex, promoting G2 arrest [47]. Down-regulation of *cyclinB2* (*CCNB2*) and *CDK6* was consistent with cell-cycle arrest, mediated by the up-regulation of *CDKN1A*, *SFN* and *GADD45*, in response to UV damage. *CDKN1A* is also able to inhibit PCNA-dependent DNA replication directly, by its direct interaction with PCNA and thus prevention of polymerase delta activation by PCNA during the S phase [49].

UV-mediated apoptosis also involves p53 signaling, with up-regulation of genes *PMAIP1* (*Noxa*) and *CYCS* (*Cytochrome C*) promoting the mitochondrial pathway of apoptosis [50]. The mitochondrial apoptotic pathway is further sustained by the down-regulation of Bcl2, reducing cellular resistance to apoptosis [50]. Again, ROS-mediated apoptosis mediated by the JAK-STAT pathway may be accompanied by the up-regulation of *SOCS* genes [51], [52]; indeed, *SOCS3* mRNA has previously been shown to be stabilized by the activation of the p38 MAPK pathway [53]. The p38 pathway also participates in the apoptotic response, by mediating the down-regulation and degradation of the phosphorylated TP63 transcription factor, which binds p53 promoter DNA [16]. Using an original approach, we could show that *TP63*, *SFN* and *CDKN1A* mRNA levels are modulated in response to UV in a p38-dependent manner. We also identified additional p38-dependent genes involved in the inflammatory response (*AREG*, *HBEGF*) [54]. This adds to growing evidence of the involvement of the p38 pathway in the UV response. Thus, although p53 plays a key role in mediating the UV response, the role of the stress-responsive MAP kinase p38 may be just as important, forming part of and strengthening the p53- mediated response to UV. Further *ex vivo* studies, coupled to whole-genome analyses, would be useful to elucidate in more detail the physiological role of the p38 pathway in the epidermal response to UV.

## Materials and Methods

### Volunteers

We recruited ten healthy female volunteers, with skin phototype II or III according to the Fitzpatrick classification [26], who had been referred for abdominal plastic surgery to the plastic-surgery department of South Hospital, Rennes, France. None of the patients had received UV radiation during the previous two months, or had taken photosensitive compounds. Patients on medical treatment or with striae on the region of the skin to be excised were excluded from the study. Each volunteer was fully informed of the procedures and gave written consent prior to taking part in the photo-biological study, which was carried out in the dermatology department of the Pontchaillou University Hospital, Rennes. The Ethics Committee of Rennes Hospital approved the study (CCPPRB N°04/36-517).

### Skin radiation and punch biopsies

A UV polychromatic light source (Dermolum UM-W1, Müller Elektronik®, Moosinning, Germany) was used, equipped with a Schott WG 305 filter to generate solar-simulated radiation (SSR) containing 5% UVB and 95% UVA. The simulated radiance was 100 mW/cm^2^ (Müller Elektronik® dosimeter). The abdomen was exposed to SSR at 2 and 4 J/cm^2^ five hours before plastic surgery to allow sufficient time to detect significant UV-induced changes in gene transcription. A dose of 2 J/cm^2^ was chosen because it corresponded to the minimal erythemal dose (MED) of SSR for phototype II Caucasian skin. Epidermis skin samples (0.5 cm Ø) from UV-irradiated areas were collected immediately after abdominal surgery and transferred directly into RNA*later* (Qiagen) for RNA extraction. Non-irradiated skin samples were taken as controls. Skin punch biopsy samples (1 cm Ø) were taken from the remaining (non-irradiated) operational area of skin from the donors to test the UV response *ex vivo*. Whole skin discs were immediately placed into wells, at the air/liquid interface of 24-well dishes containing 0.3 ml of MCF medium composed of basal medium for fibroblast culture (Biopredic International®, Rennes, France), supplemented with 2 mM L-Glutamine and 2% fetal bovine serum (FBS) (GIBCO). Prior to UV irradiation, whole skin discs were incubated at 37°C in a humidified incubator containing 5% CO_2_ for 24 hours in MCF medium containing either 0.1% DMSO or 10 µM SB203580. Skin discs were then irradiated with 2 or 4 J/cm^2^ SSR and maintained at 37°C for either 5 or 24 hours. Immediately after incubation, the epidermis section of the skin discs were cut into small fragments and stored in RNA*later* at −20°C.

### Total RNA extraction

Skin epidermis was homogenized in a Precellys®-24 device (Bertin), using ceramic beads (1.4 mm Ø, CK14), in the presence of 350 µl lysis buffer (RA1 Macherey-Nagel) supplemented with 3.5 µl β-mercapto-ethanol. The device was set at a speed of 6300 rpm, with a cycle duration of 23 seconds and an interval time between 2 cycles of 2 minutes, at 4°C. Six cycles were required for complete homogenization. Tri-reagent (400 µl; Sigma) was added, followed by 150 µl chloroform. The aqueous phase was recovered, mixed with 500 µl of 70% ethanol and transferred to a NucleoSpin® RNA II column. RNA was recovered from the column following the manufacturer's instructions, although the wash volumes were larger (RA2 = 600 µl; RA3 = 500 µl). Recovered RNA was quantified using a Nanodrop 1000 spectrophotometer (Nanodrop Technology®, Cambridge, UK) and RNA integrity was assessed using a 2100 Bioanalyser (Agilent, Palo Alto, CA, USA).

### Microarray

Experiments were performed using 44K Human Whole-Genome 60-mer oligo-chips (G4112A, Agilent Technologies) (GEO platform accession GPL1708; GEO samples series GSE20062). Total RNAs (350 ng) were amplified and labeled using a two-color labeling protocol with the Low Input Linear RNA amplification kit (p/n 5184-3523), according to the manufacturer's recommendations (Agilent). Test and reference samples were labeled with Cyanine-5 and Cyanine-3 CTP, respectively (10 mM, Perkin-Elmer/NEN Life Science). The reference sample contained an equimolar pool of combined irradiated and non-irradiated samples. Cyanine incorporation was monitored using a Nanodrop ND-1000 Spectrophotometer (values were between 1.4 and 1.6 pmol/µl). Hybridization was performed using an Agilent oligonucleotide microarray *in situ* Hybridization-Plus kit, following the manufacturer's instructions. Briefly, 750 ng of test sample cRNA was mixed with 750 ng of reference sample cRNA, in the presence of target controls. This solution was subjected to fragmentation (30 min at 60°C) and then dynamic hybridization in a rotary oven (4000 rpm, 60°C, 17 h). Slides were disassembled and washed in solutions I and II, as described in the manufacturer's instructions, and dried using a nitrogen-filled air gun before scanning. Hybridized slides were scanned with the dynamic autofocus Agilent G2565BA microarray scanner.

### Data Analysis

The Agilent feature extraction software version 9.1 and the Bioconductor package LIMMA (Smyth, 2005) were used to extract and normalize the data. The slide quality was checked for background and signal homogeneity. The background signal was subtracted for each spot. Data were normalized using the loess method and scaled with the Gquantile method [55], [56].

Multi-experiment viewer v4.4 (Mev) software was used for the Significance Analysis of Microarray (SAM) method, a multiple testing statistical analysis, to identify genes that were differentially expressed in response to SSR. Two-class unpaired analyses were made, comparing non-irradiated and irradiated samples, with a median FDR (false discovery rate) of 0%. Hierarchical Clustering (HC) and Principal Component Analysis (PCA) were used to classify the data.

### Identification of enriched biological themes

The EASE (Expression Analysis Systematic Explorer, http://david.abcc.ncifcrf.gov/tools.jsp) application was used to identify gene categories in which differentially expressed genes from the SAM analysis gene lists were over-represented. EASE uses the gene ontology (GO) systems for the categorization of genes. Gene ontology groups with an FDR score less than 0.05 and an enrichment score ≥1.5 were considered to be statistically significant.

The DAVID Pathway Viewer was used for viewing a “dynamic-gene-on-static-picture”. It was used to visualize dynamically genes of interest on manually drawn (KEGG) pathways.

We used Ingenuity Pathways Analysis (IPA) software to identify the biological mechanisms, pathways and functions most closely associated with the experimental dataset selected (http://analysis.ingenuity.com).

### RT-qPCR

Reverse transcription was performed with 1 µg of total RNA using a High-Capacity cDNA Archive Kit (Applied Biosystems) according to the manufacturer's recommendations. qPCR was performed in sealed 384-well microtiter plates using the SYBR Green TM PCR Master Mix (Applied Biosystems) with the 7900HT Fast Real-Time PCR System (Applied Biosystems). Relative amounts of transcripts were determined using the delta Ct method [57]. For each experiment, mRNA levels are expressed as fold increase following stimulation, relative to non-irradiated samples, and normalized to ribosomal *18S* transcript levels. Each experiment was carried out at least twice, and qPCR was performed in triplicate for each time point. Forward and reverse primers were designed using Primer3 software (http://frodo.wi.mit.edu) and have been tested previously for their efficiency (sequences available on request). Validated primers for *cyp2R1* were provided by Biopredic International (Rennes, France).

## Supporting Information

Table S1 Genes UP regulated/Genes DOWN regulated.
(0.03 MB XLS)

Table S2 Percentage of the total number of genes per chromosomes.
(0.02 MB XLS)

Table S3 UP-regulated genes GO terms/DOWN-regulated genes GO terms.
(0.03 MB XLS)

Table S4 T-Test values.
(0.01 MB XLS)
